# Decreased expression levels of *Ifi* genes is associated to the increased resistance to spontaneous arthritis disease in mice deficiency of IL-1RA

**DOI:** 10.1186/s12865-016-0163-y

**Published:** 2016-08-02

**Authors:** Xiaoyun Liu, Yan Jiao, Yanhong Cao, Nan Deng, Yonghui Ma, Karen A. Hasty, Andrew Kang, Hong Chen, John M. Stuart, Weikuan Gu

**Affiliations:** 1 Mudanjiang Medical College, Mudanjiang, HeilongJiang 157001 People’s Republic of China; 2 Department of Orthopaedic Surgery and Biomedical Engineering, University of Tennessee Health Science Center (UTHSC), Memphis, TN 38163 USA; 3 Center of integrative research, The first Hospital of Qiqihaer City, 30 Gongyuan Road, Longsha District, Qiqihaer, Heilongjiang 161005 People’s Republic of China; 4 Institute of Kaschin-beck Disease, Center for Endemic Disease Control, Chinese Center for Disease Control and Prevention, Harbin Medical University, Harbin, 150081 China; 5 Key Laboratory of Etiologic Epidemiology, Education Bureau of Heilongjiang Province & Ministry of Health (23618104), Harbin, 150081 China; 6 Department of Medicine, University of Tennessee Health Science Center, Memphis, TN 38163 USA; 7 Research Service, Veterans Affairs Medical Center, 1030 Jefferson Avenue, Memphis, TN 38104 USA

## Abstract

**Background:**

The mouse strain BALB/c deficient in IL-1 receptor antagonist protein (*Il-1ra*) develops spontaneous arthritis disease (SAD) while the strain DBA/1 *IL1rn*^-/-^ with the same deficiency does not. Previously, we mapped a QTL on chromosome 1 for SAD and then developed a congenic mouse strain BALB.D1-1^-/-^ that contains the QTL genomic fragment associated with resistance from DBA/1^-/-^ on a BALB/c^-/-^ background. The congenic strain was relatively resistant to spontaneous arthritis and had delayed onset and reduced severity of disease. We obtained whole genome expression profiles from the spleen of the congenic strain BALB.D1-1^-/-^ and four other strains, the wild type BALB/c, DBA/1 and the deficient DBA/1 *IL1rn*^-/-^ and the BALB/c *IL1rn*^-/-^. We then compared the similarities and differences between the congenic strain and the four parental strains. Here we report the selected potential causal genes based on differential expression levels as well as function of genes.

**Results:**

There is a considerable number of genes that are differentially expressed between the congenic strain and the three parental strains, BALB/c, DBA/1, and DBA/1^-/-^. However there only a few differentially expressed genes were identified by comparing the congenic strain and the BALB/c^-/-^strain. These differentially expressed genes are mainly from T-cell receptor beta chain (*Tcrb*) and interferon-activatable protein (*Ifi*) genes. These genes are also differentially expressed between congenic strain and BALB/c strains. However, their expression levels in the congenic strain are similar to that in DBA/1 and DBA/1^-/-^. The expression level of *Tcrb-j* gene is positively associated with two genes of *Ifi* gene 200 cluster.

**Conclusions:**

Decreased expression levels of *Ifi* genes is associated to the increased resistance to spontaneous arthritis disease and with down regulation of expressions of *Tcrb* genes in the mouse congenic strain. *Ifi* genes may play an important role in the susceptibility to SAD in mice.

**Electronic supplementary material:**

The online version of this article (doi:10.1186/s12865-016-0163-y) contains supplementary material, which is available to authorized users.

## Background

Identification of genes that regulate susceptibility to arthritis is essential in the selection of molecular targets for therapeutic application. The primary locus regulating collagen induced arthritis (CIA) was found to be within the major histocompatibility complex (MHC) loci [1–5]. This was expected because it was established that CIA in both rats and mice is MHC-linked [6–10]. Tremendous research has been done on the study of molecular mechanism of CIA model [11–15]. However, a considerable contribution to the arthritis is caused by a non-MHC-linked molecular mechanism [16–23]. Many non-MHC-linked loci have been identified [19, 22, 23], yet the causal genes for these QTL have not been known. Understanding of genes that regulate susceptibility to non-MHC-linked arthritis is essential for—the selection of molecular targets for therapy for non MHC-linked arthritis.

BALB/c and DBA/1 are important pairs of mouse strains. BALB/c and DBA/1 and C57B6/J mice share the same MHC (H-2d) haplotype; however, at the whole genome level they are genetically distant [17, 24]. In the mouse model, with a standard protocol, CIA is induced in DBA/1 and B6 [3, 10, 11, 25]. Under the same standard protocol, CIA could not be induced in BALB/c mice. The spontaneous arthritis (SAD) occurs in interleukin-1 (IL-1) receptor antagonist (IL-1rn) -deficient mice, which is dependent on non-MHC genetic bases [25, 26].

Over the last decade, we have been studying SAD in *IL-1ra*-deficient mice [25–29]. To better understand the pathogenesis of SAD, initially we used classical genetic techniques and bred susceptible and resistant mice to obtain an F2 generation and identified QTL associated with arthritis susceptibility [26]. We obtained evidence for potential QTL on chromosomes 1, 6, 11, 12, and 14. The QTL on the chromosome 1 was the major regulator for the SAD. To confirm the importance of the QTL and to identify potential candidate genes within it, we conducted speed congenic breeding to transfer the QTL region from DBA/1 mice that are resistant to spontaneous arthritis into BALB/c^-/-^ which are susceptible [29]. Our congenic breeding was successful in identifying a QTL associated with the development of spontaneous arthritis. When a fragment of DNA from the DBA/1 strain was introduced onto a BALB/c background, arthritis was delayed in onset and was less severe. Using the congenic strains that we developed, the genomic region of the originally identified QTL was redefined into a region that is downstream from the peak region of our original mapping [26, 27, 29].

Microarrays have been used for the analysis of whole genome expression profiles for the more than a decade. They have developed as a mature technology not only in the producing of gene expression profiles but also in the analysis of differentially expressed genes. One of the useful applications of microarray-generated whole genome expression profiling is the identification of candidate genes that regulate a specific disease trait or a molecular pathway [28, 30–34].

According to the Ensembl database, the transferred region from DBA/1-/- to BALB/c-/- is between D1Mit110 and D1Mit209 on chromosome 1 with a size of 23.73 Mb which contains 320 genetic elements. Among those genes, 115 are identified as genes relevant to arthritis and its potential pathways [29]. Although the number of genes seems large and to some degree difficult to specifically target candidate genes, comparison of the data from congenic strains provide a defined genomic region for their localization. The use of congenic strains enable us to analyze the effect of the transferred fragment within QTL region on global gene expression. Previously, we have been using the microarray tools for the study of differential gene expression comparing diseased knockout and normal wild type mice [28, 32, 33]. In this study, we compared gene expression profiles of the whole genome of the congenic strain and parental strains.

## Methods

### Mice

Female mice at 4 months of age from a congenic strain BALB.D1-1, BALB/c and DBA/1 wild type, and BALB/c and DBA/1 Il1rn knockout mice (Table 1) [29] were used to generate gene expression data using the Illumina platform. The strain BALB.D1-1 is on the BALB/c genomic background contains genetic DNA from DBA/1 between D1Mit55 and D1MitD1Mit209 inclusive. Analysis of each strain was done with three spleens, each from a different female mouse. All mice have been maintained in the animal facility of the Department of Veterans Affairs Medical Center, Memphis. Experimental animal procedures and mouse husbandry were performed in accordance with the National Institutes of Health’s Guide for the Care and Use of Laboratory Animals and approved by the VAMC Institutional Animal Care and Use Committee.

**Table 1:** Mouse strains used in this study

Strain	IL-1 Receptor Antagonist	Description
BALB/c	+/+	Normal strain originally from the Jackson laboratory
DBA/1	+/+	Normal strain originally from the Jackson laboratory
BALB/c^-/-^	-/-	With mutation of *IL1rn*
DBA/1^-/-^	-/-	With mutation of *IL1rn*
BALB.D1-1	-/-	With mutation of *IL1rn,* with a piece of fragment from DBA/1, with BALB/c background.

### Genotyping

The insertion and genotype of the congenic BALB.D1-1 mice was confirmed before RNA extraction and microarray analysis [26]. Genomic DNA was extracted from tissues obtained by ear punch. The procedure used has been previously described [20]. Briefly, DNA was extracted from the tissue and amplification of microsatellite markers conducted by polymerase chain reaction (PCR). PCR products were analyzed using poly-acrylamide gel electrophoresis using the Mega-Gel Dual High-Throughput Vertical Electrophoresis System (C.B.S. Scientific, Del Mar, CA).

### RNA extraction

RNA was extracted from spleens using a Trizol reagent (Invitrogen, CA). Total RNAs were purified using the RNeasy MinElute Cleanup Kit (Qiagen, CA). RNA quality and integrity were analyzed by the Agilent Bioanalyzer [33].

### Microarray procedure

A starting amount of 200 ng of high-quality total RNA, with a RIN (RNA Integrity Score) number of more than seven, was used to generate cDNA and cRNA using the Illumina® TotalPrep™ RNA Amplification Kit (Ambion). For each of five individual samples, 1.5 ug of cRNA sample was hybridized overnight to Illumina mouse-6 v1.1 expression beadchips in a multiple step procedure according to the manufacturer’s instructions; the chips were washed, dried and scanned on the BeadArray Reader (Illumina, CA) and raw data were generated using BeadStudio 2.3.41 (Illumina, CA) [28, 33].

### Analysis of microarray data

Raw data were normalized with quantile methodology using BeadStudio software. Four comparisons were made. The gene expression profiles of BALB.D1-1 compared to that of BALB/c^-/-^, BALB/c wild type, DBA/1^-/-^, and DBA/1 wild type. The Diff Score from the quantile method is a transformation of the p-value that provides directionality to the p-value based on the difference between the average signal in the reference group vs. the comparison group. For a p-value of 0.05, DiffScore = ± 13; For a *p*-value of 0.01, DiffScore = ± 22; For a *p*-value of 0.001, DiffScore = ± 33. The DiffScore in our initial analysis was set up as ± 10, to ensure we will not miss the potential candidate genes [33, 35].

### Candidate and pathway analysis and construction

To identify candidate genes and construct pathway/connections, we correlated expression between candidate genes within QTL region and Il1rn, including Il1a, Il1b, Il1r1, Il1r2. For pathway analysis, we took full advantage of existing data of gene expression profiles at GeneNetwork at http://www.genenetwork.org/webqtl/main.py. We used gene expression data from spleens obtained from a set of recombinant inbred (RI) strains derived from C57BL/6 J (B6) and DBA/2 J (D2) [18, 36]. Data was generated using the Affymetrix GeneChip Mouse Gene 1.0 ST array (http://www.genenetwork.org/webqtl/main.py?FormID=sharinginfo&GN _AccessionId = 283). In the case of multiple probes, the one with highest expression level was chosen for the analysis [37]. The gene networks were constructed using application tools in GeneNetwork. We constructed the gene network based on the Network Graph in combination with the Correlation Matrix. Network graph is often used to visualize multiple sets of interactions. The Spring Model layout (force reduction) was used for the graphic method for all graphic samples. We followed standard criteria for the strong, correlation, correlation, and none correlation. When the R value is equal or more than 0.7 or -0.7, we regard the correlation is strong positive or negative correlation. When the R value is between 0. 5 and 0.69 or -0. 5 and -0.69, the correlation exists but not as strong. Any R value between 0 and 0.5 or 0 and -0.5 is treated as none-to-weak-correlation [33, 35]. Initial analysis of relevance of genes to arthritis was conducted using PGMapper [37].

## Results

### Similarities of whole genome gene expression profiles among different strains

**Fig. 1:**
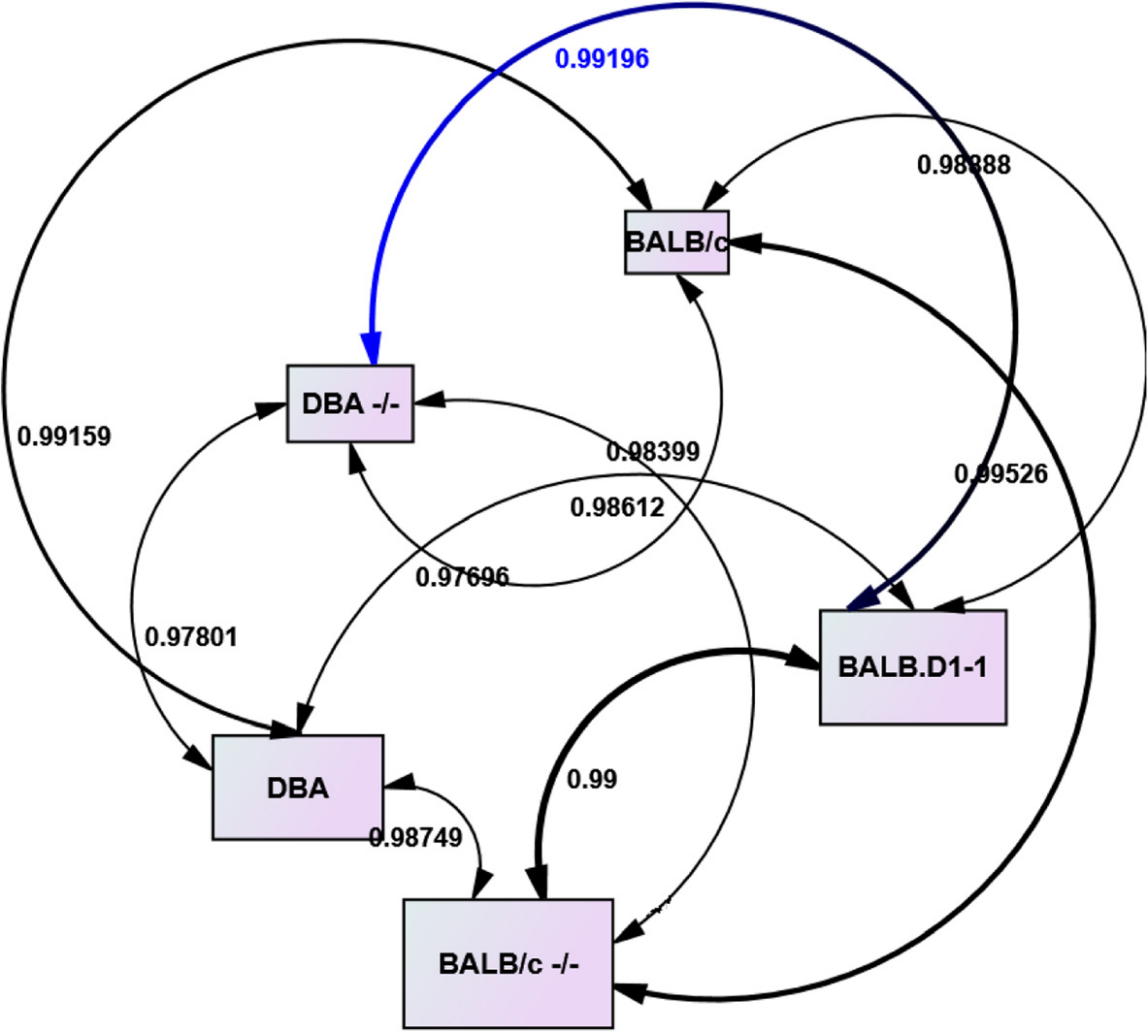
Correlation between among BALB.D1-1 and four strains. Strain names are listed on the left vertical bar. R values are listed under the horizontal bar. The gene expression profile of wild type DBA/1 (DBA) has the lowest similarity with that of congenic mice, which has a mutation of Il1rn and is on the BALB/c genomic background.in the square boxes. Double-arrow lines are used to connect the two strains in comparisons. Correlation R values are listed along the arrow lines

The congenic strain BALB.D1-1 contains a genomic fragment flanked by markers D1Mit55 and D1Mit209 from DBA/1 in BALB/c background [20]. We first examined the similarities of whole genome gene expression of BALB.D1-1 to the other four strains of mice. The Illumina mouse-6 v1.1 expression beadchip contains 30774 probes (46632 elements) representing 29940 mouse genes. We calculated the R (Correlation) of expression levels of probes between BALB.D1-1 and the other four strains (Fig. 1). Although R values to those four strains indicates that the expression profiles of BALB.D1-1 is significantly related to all of those four strains, there are slight differences among them. Because the BALB.D1-1 is deficient in IL-1 receptor antagonist protein (IL-1RA), its expression profile is more similar to the two knockout strains, ^-/-^DBA/1^-/-^and BALB/c^-/-^, than to the wild type strains, BALB/c and DBA/1. Because of the insertion of a fragment from DBA/1, BALB.D1-1 acts similar to ^-/-^DBA/1^-/-^, showing resistance to spontaneous arthritis [20] with a gene expression profile resembling the ^-/-^DBA/1^-/-^. However, because BALB.D1-1 is on the BALB/c background, it is least related to DBA/1 with the R value of 0.976964.

### Gene expression profiling between BALB.D1-1 and DBA/1 wild type

Because the BALB.D1-1 is under the BALB/c background with the spall piece of fragment from DBA background, one would expected to see the similarity of a small number of genes in the expression levels. However, the expression levels of more than 97% of genes between these two strains are similar. We then focused on the genes in the QTL region. We found that all of the genes in this region are expressed at the similar level between these two strains.” In particular, we noticed that all of the genes in the interferon activated (*Ifi*) gene family are similar between these two strains.

**Fig. 2:**
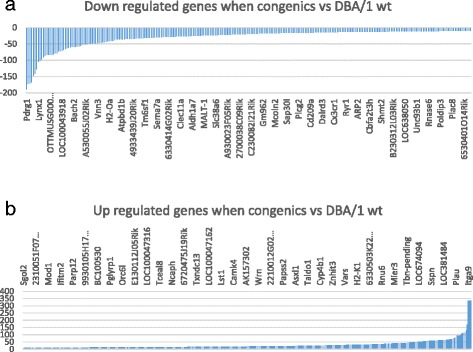
Up and down regulated genes and networks in congenic mice in comparison to that of DBA wild type mice. **a** Down regulated genes in congenic mice. Probe/gene names are shown above the horizontal bar. Numbers on the left of vertical bar is the score of differential expression. **b** Up regulated genes in congenic mice. Cut off score as significant is -2 or 2. . Probe/gene names are shown above the horizontal bar. Numbers on the left of vertical bar is the score of differential expression

Compared to the expression levels of DBA/1, the expression levels of 264 probes were down regulated and 472 probes were up regulated in the BALB.D1-1 (Fig. 2a, b). Among the 472 up regulated probes, 344 are known genes. By searching the PubMed using PGMapper, we found that, among the 344 known genes, 61 are arthritis relevant. Two genes represented by those 472 probes are located in the QTL region, *Darc* and *Fcgr4* (Additional file 1: Table S1). Among the 264 down regulated probes, three are within the QTL region, *Kmo, Fcrla*, and *Ephx1*. Probes of 189 out of 264 represent known genes. Among those known genes, 26 are categorized by PGMapper as arthritis relevant genes (Additional file 1: Table S1).

The 61 up regulated and 26 down regulated genes together with Il1a, Il1b, Il1r1, Il1r2, Il1rap, Il1rapl1, Il1rn are analyzed at Genenetwork. Using UTHSC Affy MoGene 1.0 ST Spleen (http://www.genenetwork.org/webqtl/main.py?FormID=sharinginfo&GN_AccessionId=283) were identified 162 probes. After eliminating some duplicate probes, 138 probes were used for the gene network construction [38]. The gene Network shows that none of these genes directly connect to the Il1rn or other interleukin 1 family genes (Additional file 2: Figure S1).

Thus, none of the genes that showed differential expression are likely candidate genes that lead to the increased resistance to SAD.

### Gene expression profiling between BALB.D1-1 and ^-/-^DBA/1^-/-^

Both BALB.D1-1 and ^-/-^DBA/1^-/-^ are lack of *IL1rn* gene. Their gene expression levels showed more similarity in comparing to that between BALB.D1-1 and ^-/-^DBA/1. We confirmed that the expression levels of *Ifi* gene family are similar between these two strains.

**Fig. 3:**
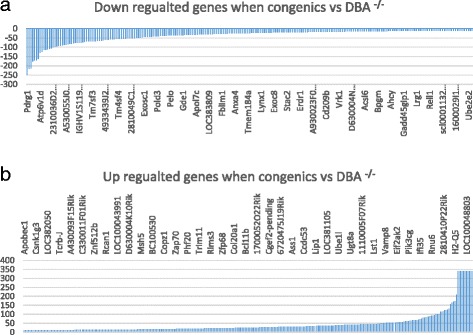
Up and down regulated genes and networks in congenic mice in comparison to that of ^-/-^DBA/1^-/-^mice. **a** Down regulated genes in congenic mice. Probe/gene names are shown above the horizontal bar. Numbers on the left of vertical bar is the score of differential expression. **b** Up regulated genes in congenic mice. Cut off score as significant is -2 or 2. . Probe/gene names are shown above the horizontal bar. Numbers on the left of vertical bar is the score of differential expression

We then conducted further comparison to eliminate the possibility of other genes as favorite candidate genes. Compared to the expression levels of DBA/1^-/-^, the expression levels of 241 probes were down regulated and 310 probes were up regulated in the BALB.D1-1 (Fig. 3a, b). Among the 310 up regulated probes, 235 are from known genes (Additional file 3: Table S2). Searching PubMed with PGMapper on Oct 2, 2013, 43 of those known genes were found to be relevant to arthritis. Among the 241 down regulated probes, 212 are from known genes (Additional file 3: Table S2). Searching PubMed with PGMapper on Oct 2, 2013, 29 of those known genes were found to be relevant to arthritis. A total of 138 probes for the 43 up and 29 down regulated and the seven Il1rn family genes were identified from the Using UTHSC Affy MoGene 1.0 ST Spleen. After eliminating some duplicate probes, 101 probes were used for the analysis [28]. Similar to that of DBA wt mice, data analyses of gene networks indicate that there is no strong connection of expression levels between these genes and Il1rn family. (Additional file 2: Figure S2).

### Gene expression profiling between BALB.D1-1 and BALB/c

**Fig. 4:**
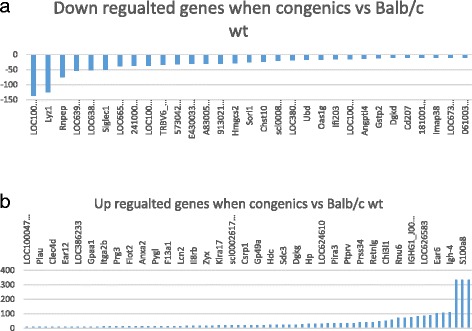
Up and down regulated genes and networks in congenic mice in comparison to that of BALB/c wild type mice. **a** Down regulated genes in congenic mice. Probe/gene names are shown above the horizontal bar. Numbers on the left of vertical bar is the score of differential expression. **b** Up regulated genes in congenic mice. Cut off score as significant is -2 or 2. . Probe/gene names are shown above the horizontal bar. Numbers on the left of vertical bar is the score of differential expression

Compared to the expression levels of BALB/c, the expression levels of 31 probes were down regulated and 70 probes were up regulated in the BALB.D1-1 (Fig. 4a, b). Among the 70 up regulated probes, all are from known genes. One gene was found in QTL region, *Ifi202b*. Among 31 down regulated probes, 30 are from known genes. Comparing to genes in QTL region, one gene is in the list of the QTL region, *Ifi203* (Additional file 4: Table S3). The 70 up regulated and 30 down regulated genes were analyzed using PGMapper and Siglec1 was found to relevant to arthritis. The 128 probes were identified to represent the 70 and 30 up and down regulated genes respectively and the Ilrn family. After removing the duplicate probes, 92 probes were used for the gene network construction. The gene network indicated that that none of these genes directly connected to the Il1rap or other interleukin 1 family genes (Additional file 2: Figure S3).

### Gene expression profiling between BALB.D1-1 and BALB/c^-/-^

**Fig. 5:**
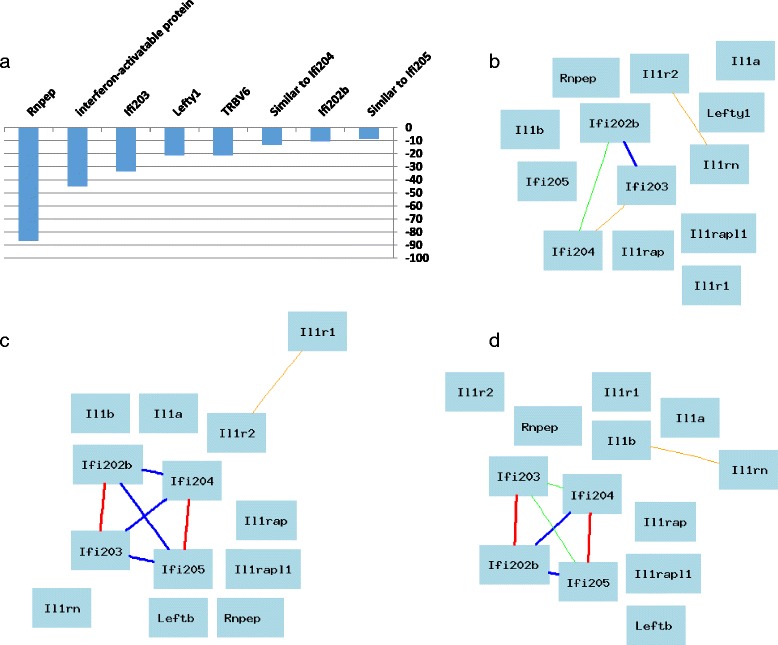
Down regulated genes and network in congenic mice in comparison to that of BALB/c^-/-^type. **a** Down regulated genes in congenic mice. Probe/gene names are shown above the horizontal bar. Numbers on the left of the vertical bar is the score of differential expression. **b** Network of known arthritis relevant down regulated genes when congenics are compared to BALB/c^-/-^based on gene expression profiles in the spleen. The 13 nodes in the graph below show the selected traits. All nodes are displayed. The 4 edges between the nodes, filtered from the 78 total edges and drawn as curves, show Pearson correlation coefficients greater than 0.5 or less than -0.5. The graph’s canvas is 40.0 by 40.0 cm, and the node labels are drawn with a 16.0 point font, and the edge labels are drawn with a 16.0 point font. **c** Network of known arthritis relevant down regulated genes when congenics vs BALB/c^-/-^based on gene expression profiles in T help cells. The seven edges between the nodes, filtered from the 78 total edges and drawn as curves, show Pearson correlation coefficients greater than 0.5 or less than -0.5. **d** Network of known arthritis relevant down regulated genes when congenics are compared to BALB/c^-/-^based on gene expression profiles in T cell regulatory cells

**Fig. 6:**
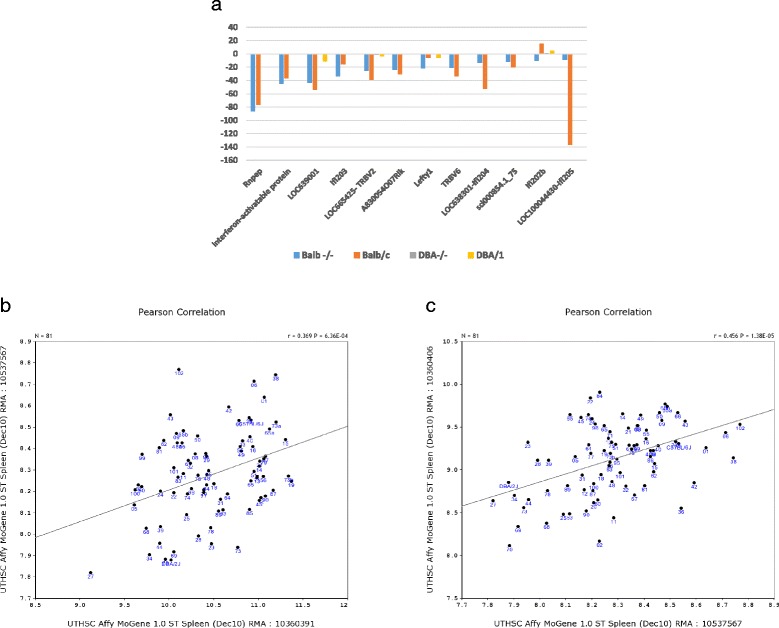
Involvement of interferon-activatable protein and T-cell receptor beta chain genes in the resistance to SAD. **a** Gene expression levels of congenic strain in comparing with four other strains. Numbers on the left vertical bar are fold changes. Names of probes/genes are listed below the figure on horizontal axis. Four different strains are labeled with different colors. Expression levels of genes in congenic strain are down regulated in comparing with that of BALB^-/-^ and BALB/c but not significant difference to the other two strains, the ^-/-^DBA/1^-/-^ and DBA/1. **b** Positive correlation of gene expression levels between *Ifi*203 (probe 10360391) and one probe for T-cell receptor beta, joining region (Tcrb-j) (Probe ID: 10537567) from the whole genome profiles of mouse spleen in BXD strains. Numbers on the left vertical bar are the relative expression level of Tcrb-j of an individual. Names listed below the figure on horizontal axis are the relative expression levels of *Ifi*203 of an individual. **c** Positive correlation of gene expression levels between *Ifi*205 (probe 103603406) and Tcrb-j (probe 10537567). Numbers on the left vertical bar are the relative expression level of Tcrb-j of an individual. Names listed below the figure on horizontal axis are the relative expression levels of *Ifi*205 of an individual

Compared to the expression levels of BALB/c^-/-^, the expression levels of 12 probes were down regulated, but no probes were significantly up regulated in the BALB.D1-1 (Fig. 5a). Among the 12 down regulated probes, 8 are from the known genes or sequences, LOC100040462 (*Mndal*), LOC639001 (T-cell receptor beta chain V region C5 precursor), *Ifi203*, LOC665425 (T-cell receptor beta chain V region LB2 precursor), *Lefty1*, *Trbc6*, LOC638301, *Ifi204, Ifi202b* (Fig. 6a). Based on the search of results from PubMed with PGMapper, none of those known genes were found to be relevant to arthritis. The interferon activated gene family (*Ifi202b, Ifi203, Ifi204, Mndal*) and *Lefty1* are located in QTL region.

Six of the 11 probes (*Rnpep*, *Ifi203*, *Ifi204*, *Ifi205*, *Lefty1*, *Ifi202b*) were found in the UTHSC Affy MoGene 1.0 ST Spleen database. Those 6 probes are analyzed together with *Il1a, Il1b, Il1r1, Il1r2, Il1rap, Il1rapl1, Il1rn* at Genenetwork. Using the whole genome expression profile from the spleen, we constructed the network of these genes (Fig. 5b), which shows that there is no strong correlation between the expression levels of *IL1rn* family and the interferon-activatable protein (*Ifi*) family members. We then constructed the gene network using whole genome expression profiles from the HZI T-Helper Cell Affymetrix M430v2 (http://www.genenetwork.org/webqtl/main.py?FormID=sharinginfo&GN_AccessionId=319) and HZI T-regulatory T cells (CD4 + CD25+) (http://www.genenetwork.org/webqtl/main.py?FormID=sharinginfo&GN_AccessionId=122). Data for gene networks of both T helper cells and T regulatory cells support that there is no strong connection between the expression levels of *IL1rn* family and the *Ifi* family members (Fig. 5c and d). Even after a further decrease of the threshold for significance to 0.35, there still no direct correlation between *IL1rn* and the *Ifi* family members.

### Potential involvement of interferon-activatable protein and T-cell receptor beta chain genes in the resistance to SAD

We next compared the expression levels of all the 12 down regulated genes through the comparison between the congenic strain and the four parental strains above. As shown in Fig. 6, 11 of 12 down regulated probes in the congenic strain, compared to BALB^-/-^mice, were also down regulated when compared to that of BALB/c wt. None of the genes were down regulated when compared to that of ^-/-^DBA/1^-/-^and DBA/1 wt. These data means that the expression levels of these genes are high in the BALB/c and BALB^-/-^while the expression levels of these genes are low in DBA/1 wt and ^-/-^DBA/1^-/-^. Considering the fact that ^-/-^DBA/1^-/-^is resistant to SAD while BALB^-/-^is susceptible to SAD, the decreased expression level of these genes in the congenic stain suggests that the increased resistance in congenic mice may be due to the down regulation of these genes.

Because T-cell receptor beta chain genes are not located on mouse chromosome 1. They are not the causal genes for the QTL. The only other group of differential expressed genes are the genes of *Ifi* cluster. If the genes of *Ifi*200 cluster is the causal genes for the QTL, then the decreased expression level of T-cell receptor beta chain genes should be caused by the decreased level of genes of *Ifi*200 cluster. Thus, there should be positive correlation at gene expression levels between at least one T-cell receptor beta chain gene and at least one gene in the *Ifi*200 cluster. We then identified probes for *Ifi* 200 cluster and one probe for T-cell receptor beta, joining region (Tcrb-j) from the whole genome profiles of mouse spleen in BXD strains. Our analysis shows that the expression levels of *Ifi*203 and *Ifi*205 are positively associated to that of Tcrv-j (Fig. 6b and c). Thus, in the case of congenic strain, the decreased expression levels of *Ifi*203 and *Ifi*205 may lead to the decreased expression levels of T-cell receptor beta chain genes. In turn, the down regulation of expression levels of T-cell receptor beta chain genes may lead to the increased resistance to spontaneous arthritis disease in the mouse congenic strain.

### Real-time PCR for confirmation of expression level of *Ifi*203

**Fig. 7:**
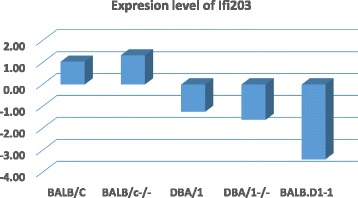
The expression level of *Ifi*203 in spleens of five mouse strains. Strain names are shown on the horizontal bar. Numbers on the left of vertical bar is the score of level of relative expression. The expression level of *Ifi*203 in DBA/1, DBA1/-/- and congenic strains are low while the in BaLB/c and BALB/c-/- are high

Although our laboratory has been conducting microarray analysis for the past 13 years and we have eliminated all possible factors that may lead to errors or variations of data, we have conducted real time qPCR to confirm the microarray data using *Ifi*203 as one of the key genes in this study. AS shown in Fig. 7, the expression level of *Ifi*203 in BALB/c and BALB/c-/- are high while in DBA/1 and DBA/1-/- are low. In BALB.D1-1, its expression level is the lowest among these strains and is much lower than that in BALB/c and BALB/c-/-. This data confirms that the decreased expression level of *Ifi*203 in BALB.D1-1 detected by microarray.

## Discussion

Our multiple comparisons confirmed that the decreased expression levels of genes in T-cell receptor beta chain and *Ifi* cluster in the congenic strain are the major change that potentially leads to the increased resistance to SAD in the congenic strain in comparing to that of BALB/c^-/-^. The expression levels of these genes are similar to that in DBA and DBA/1^-/-^, which are resistant to the SAD. The expression of these genes is lower than that of BALB/c and BALB/c^-/-^, which are susceptible to SAD. There are considerable number of genes that are differentially expressed between the congenic strain and three other parental strains, the DBA, ^-/-^DBA/1^-/-^and BALB/c because of the different genomic background and mutation of Il1r. However, there is only one difference between the congenic strain and BALB/c^-/-^. The difference is that in congenic mice, the genome region of QTL on chromosome 1 is replaced by the fragment from DBA while the entire remainder of the genome of BALB/c^-/-^are from the BALB/c strain. The small number of candidate genes derived from our analyses pave the way to final determine the causal gene in the congenic strain.

Our preliminary data indicated the *Ifi* gene 200 cluster contains the favorite candidate gene for the SAD resistance in the congenic strain. Among differentially expressed genes between congenic and BALB/c^-/-^, four genes in the *Ifi* gene 200 cluster are differentially expressed and are located in transferred genomic fragments in congenic strains. These genes are located in the transferred QTL genomic fragment on mouse chromosome 1, which is between D1Mit110 (167758517-167758653) and D1Mit209 (191493187-191493284). Our earlier analysis indicated that four candidate genes: *Fcer1g, Fcgr3, Ifi202b*, and *Kmo a*re among the differentially expressed genes [29, 34]. Our current analysis indicates that the other three genes, *Fcer1g, Fcgr3,* and *Kmo,* were not differentially regulated in congenic strains compared to BALB/c^-/-^ that is susceptible to spontaneous arthritis. Interferon-activatable protein has been reported as an immune suppressor [36, 38–40]. For example, *Ifi202* is known as an interferon-inducible lupus susceptibility gene [36, 38]. Silencing of *Ifi202b* in tolerized CD8+ Ti cells abrogated the suppressive capacity of these cells. Recently the role of *Ifi*204 in the response to bacterial infection has been investigated [39]. It is expected that in the near future much broad function of *Ifi* family genes in the infection and immune innate immune responses will be identified [40]. Accordingly, it is very important to further investigate their candidacy.

The regulatory role of the T-cell receptor beta chain in the susceptibility of arthritis is well known [41–43]. Current information indicates that genes of T-cell receptor beta chain are not located in the transferred genomic fragment of the QTL region in the congenic strain. However, it is important to investigate if the genes between T-cell receptor beta chain family and *Ifi* family interact to down regulate SAD in the congenic strain.

In addition to the T-cell receptor beta chain and *Ifi* genes, we also detected the differential expression of *Lefty1* in the QTL region. Early study indicated that *Lefty1*, *Lefty2*, and *Nodal* are expressed on the left side of developing mouse embryos and are implicated in L-R determination. Recently, because of the unique ability of *Lefty* to reroute the cell fate by making cells nonresponsive to diverse differentiation factors such as *Nodal* or other signals that require *Egfcfc* as a co-receptor, *Lefty* has become regarded as a determinant of cell stemness or differentiative events. There is no report for its role in arthritis, inflammation or infection. At present, we do not have enough information to explain its role in the congenic strains.

Our study was done with three replicates, each from female mice from each strain. The data may reflect a bias due to the use of female mice and may not represent that from male mice. Sex hormones have been shown to differentially regulate expression of *Ifi*202 [44]. However, all the strains are measured at the same age stge. Therefore, the differences at least reflect the genomic variation in the female mice between congenic and other strains. We used the spleen as the organ for the RNA extraction and whole genome gene profiling [28]. Although spleen as an organ important for immune, the phenotype of SAD in these mice were based on the joints. Therefore, we do not rule out that possibility that the data may not reflect what all happened in the joints. However, we believe that the molecular mechanism detected from this study is important to elucidate the causal gene for the congenic strain. Further study to directly link the candidate to the disease is necessary.

The “interferon-activatable protein” was labeled in the Illumina chip for a probe of LOC100040462. However, it lately has been validated as a transcription factor called *Mndal*, which is also a member of *Ifi* family gene. Interestingly, Zhang et al. in their paper indicated that *Mndal* is absent from DBA/2j [45]. We assume that DBA/1, the strain we used in this study, is also absent. The increased level of expression level of *Mndal* in this case perhaps represents the *Mndal* gene from absent to present. However, the expression level from the DBA/1j genome detected by this probe is above the none-expression level (Absolute value over 118) while the expression level from the Balb/c genome is twice higher than that of the DBA/1j genome. As to its potential effect, it could be to interact with other genes in the *Ifi* family or to affect the expression level of heat hsp105/110hDa and Igkc2-112, which are only two known genes which showed the decreased expression between the congenic strain and the most closest parental strain, DBA-/-. However, because of the differential expression level of other genes in *Ifi* family, whether *Mndal* affect the expression of hsp105/110hDa and Igkc2-112 need further study.

Our data indicated that many differentially expressed among those mouse strains are not associated with Il1-rn. This result is not completely unexpected. First of all, SAD occurs in the mouse strain that is lack of expression of Il1-rn. One would predict that the gene expression profile of such a mouse strain does not reflect the pathways that involves the Il1-rn. Secondly, the causal genes derived from the comparison between the congenic strain BALB.D1-1 and BALB/c-/- are under the genomic background of lack of Il1-rn. Thus, the causal genes are in fact acting when there is a lack of Il1-rn. It is important to notice that with standard protocol, CIA has been induced in DBA/1 and B6 [3–5]. Under standard protocol, CIA could not be induced in BALB/c mice without modification. The SAD in interleukin-1 (IL-1) receptor antagonist (IL-1rn) -deficient mice is dependent on non-MHC (major histocompatibility complex) genes in mice. It is well-known that Il1-rn has played an important role in the arthritis. The role of *Ifi* family genes in SAD may act in the population that is non-MHC and lack of role of Il1-rn. On the other hand, the lack of association in the gene expression does not rule out the protein-protein interaction. The protein products of these two gene families may be directly interact or binding to each other.

## Conclusions

Our study demonstrated the association of expression levels of the *Ifi* gene 200 cluster to the susceptibility of mice to SAD. Our data also suggest that the regulation of SAD by the *Ifi* gene 200 cluster is potentially through the regulation of genes of T-cell receptor beta chain.

## Abbreviations

CD4, cluster of differentiation 4; CIA, collagen-induced arthritis; *Ifi*, interferon-activatable protein; *Il-1ra*, IL-1 receptor antagonist protein; PCR, polymerase chain reaction; QTL, quantitative trait loci; SAD, spontaneous arthritis disease; *Tcrb*, T-cell receptor beta chain

## Additional files

Additional file 1: Table S1. Down and up regulated genes comparing congenic strain to that in DBA wild type. List of names and DiffScores of changes of down or up regulated genes in congenic strain in comparison to that of DBA wild type. (XLSX 26 kb) Additional file 2 Figure S1. Network of known arthritis relevant up and down regulated genes when congenics vs DBA/1 wild type. The 138 nodes in the graph below show the selected traits. Only nodes with edges are displayed. The 167 edges between the nodes, filtered from the 9453 total edges and drawn as lines, show Pearson correlation coefficients greater than 0.75 or less than -0.75. The graph’s canvas is 40.0 by 40.0 cm, and the node labels are drawn with a 16.0 point font, and the edge labels are drawn with a 16.0 point font. **Figure S2** Network of known arthritis relevant up and down regulated genes when congenics vs DBA^-/-^. The 101 nodes in the graph below show the selected traits. Only nodes with edges are displayed. The 38 edges between the nodes, filtered from the 5050 total edges and drawn as lines, show Pearson correlation coefficients greater than 0.75 or less than -0.75. The graph’s canvas is 40.0 by 40.0 cm, and the node labels are drawn with a 16.0 point font, and the edge labels are drawn with a 16.0 point font. Figure S3 Network of known arthritis relevant up and down regulated genes when congenics are compared to BALB/c wild type. The 92 nodes in the graph below show the selected traits. Only nodes with edges are displayed. The 40 edges between the nodes, filtered from the 4186 total edges and drawn as lines, show Pearson correlation coefficients greater than 0.75 or less than -0.75. The graph’s canvas is 40.0 by 40.0 cm, and the node labels are drawn with a 16.0 point font, and the edge labels are drawn with a 16.0 point font. (DOCX 71 kb) Additional file 3: Table S2. Down and up regulated genes comparing congenic strain to that of DBA Il1rn mutation. List of names and DiffScores of changes of down or up regulated genes in congenic strain in comparison to that of DBA Il1rn mutation. (XLSX 21 kb) Additional file 4: Table S3. Down and up regulated genes comparing congenic stain to the Balb/c wild type. List of names and DiffScores of changes of down or up regulated genes in congenic strain in comparison to that of Balb/c wild type. (XLSX 10 kb)
