# Supplementary figures and images for: Decreased expression levels of Ifi genes is associated to the increased resistance to spontaneous arthritis disease in mice deficiency of IL-1RA
Source: BMC Immunol. 2016 Aug 2;17:25. doi: 10.1186/s12865-016-0163-y (PMC4970213; doi:10.1186/s12865-016-0163-y)

Supplementary Figure s1.


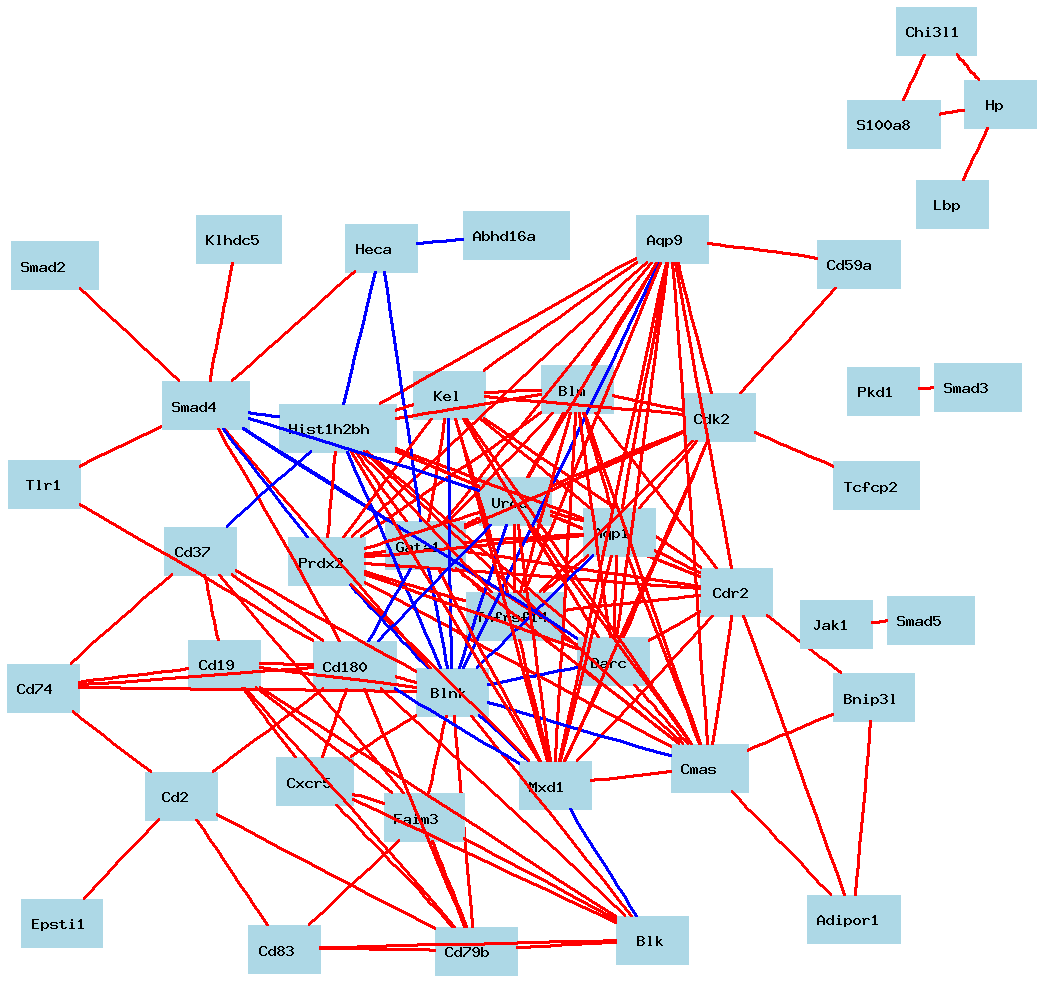


Supplementary figure S2.


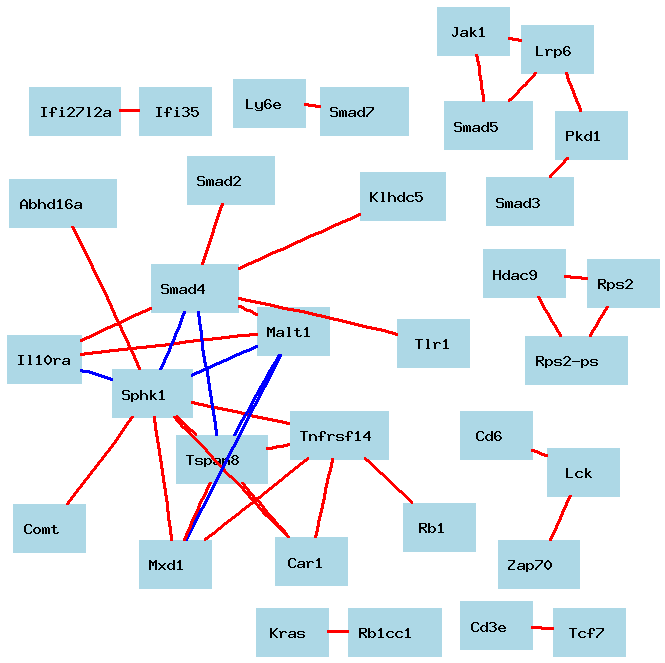


Supplementary Figure s3.


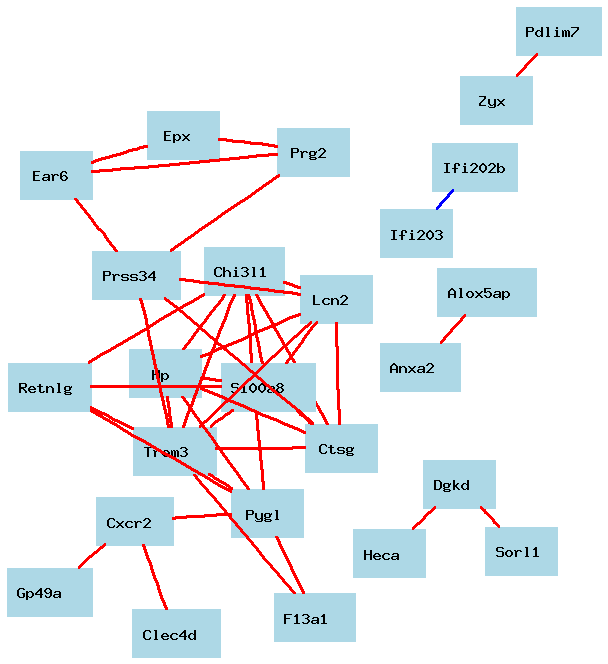

Supplement: Additional file 2 Figure S1. — Network of known arthritis relevant up and down regulated genes when congenics vs DBA/1 wild type. The 138 nodes in the graph below show the selected traits. Only nodes with edges are displayed. The 167 edges between the nodes, filtered from the 9453 total edges and drawn as lines, show Pearson correlation coefficients greater than 0.75 or less than -0.75. The graph’s canvas is 40.0 by 40.0 cm, and the node labels are drawn with a 16.0 point font, and the edge labels are drawn with a 16.0 point font. Figure S2 Network of known arthritis relevant up and down regulated genes when congenics vs DBA-/-. The 101 nodes in the graph below show the selected traits. Only nodes with edges are displayed. The 38 edges between the nodes, filtered from the 5050 total edges and drawn as lines, show Pearson correlation coefficients greater than 0.75 or less than -0.75. The graph’s canvas is 40.0 by 40.0 cm, and the node labels are drawn with a 16.0 point font, and the edge labels are drawn with a 16.0 point font. Figure S3 Network of known arthritis relevant up and down regulated genes when congenics are compared to BALB/c wild type. The 92 nodes in the graph below show the selected traits. Only nodes with edges are displayed. The 40 edges between the nodes, filtered from the 4186 total edges and drawn as lines, show Pearson correlation coefficients greater than 0.75 or less than -0.75. The graph’s canvas is 40.0 by 40.0 cm, and the node labels are drawn with a 16.0 point font, and the edge labels are drawn with a 16.0 point font. (DOCX 71 kb) [file 12865_2016_163_MOESM2_ESM.docx]
